# Editorial: Role of colchicine in atherosclerosis

**DOI:** 10.3389/fcvm.2024.1516185

**Published:** 2024-11-21

**Authors:** Ashish Misra, Peter J. Psaltis, Stefan Mark Nidorf

**Affiliations:** ^1^ Atherosclerosis and Vascular Remodeling Group, Heart Research Institute, Sydney, NSW, Australia; ^2^ Faculty of Medicine and Health, The University of Sydney, Sydney, NSW, Australia; ^3^ Vascular Research Centre, Lifelong Health Theme, South Australian Health and Medical Research Institute, Adelaide, SA, Australia; ^4^ Faculty of Health and Medical Sciences, Adelaide Medical School, The University of Adelaide, Adelaide, SA, Australia; ^5^ Department of Cardiology, Central Adelaide Local Health Network, Adelaide, SA, Australia; ^6^ Heart and Vascular Research Institute, Harry Perkins Institute of Medical Research, Perth, WA, Australia

**Keywords:** atheroscelorsis, inflammation, coronary artery diasease, IL1 beta, macropahge, colchicine

**Editorial on the Research Topic**
Role of colchicine in atherosclerosis

Colchicine, an ancient drug with a well-established safety record, that is now approved for secondary prevention of atherosclerosis based upon its proven efficacy and safety.

Atherosclerosis evolves due to the accumulation of cholesterol and inflammatory cells in the artery wall, resulting in the development of vessel-occluding plaques that can lead to chronic ischemia and risk of life-threatening cardiovascular complications, including stroke and myocardial infarction (MI) due to acute plaque rupture (1). Although commonly viewed as a condition driven predominantly by cholesterol, it has been known for over 160 years to also be driven by an inflammatory response to cholesterol that has recently been demonstrated to occur as it forms into crystals in the arterial wall. Thus, despite use of potent cholesterol-lowering agents, patients face a substantial life-time risk of recurring major adverse cardiovascular events (MACE) (2–4) due to failure to adequately dampen the inflammatory process.

Over the last decade landmark clinical trials, such as CANTOS (5), COLCOT (6) and LoDoCo2 (7), have provided strong evidence that dampening inflammation leads to better clinical outcomes in patients with atherosclerotic cardiovascular disease. Despite the effectiveness of canakinumab demonstrated in CANTOS (5), low-dose colchicine (0.5 mg daily) is currently the only FDA approved anti-inflammatory agent available for clinical use in patients with established coronary disease.

In a recent meta-analysis Zhou et al. of five large, long-term randomised trials that included >14,000 patients, it was found that long-term use of low-dose colchicine decreased both MACE and its individual components of MI, stroke and need for revascularisation in patients with established atherosclerosis. However, while it is known that colchicine has broad anti-inflammatory effects, the exact mechanisms by which it confers cardiovascular protection is still the subject of ongoing research (Figure 1).

**Figure 1:**
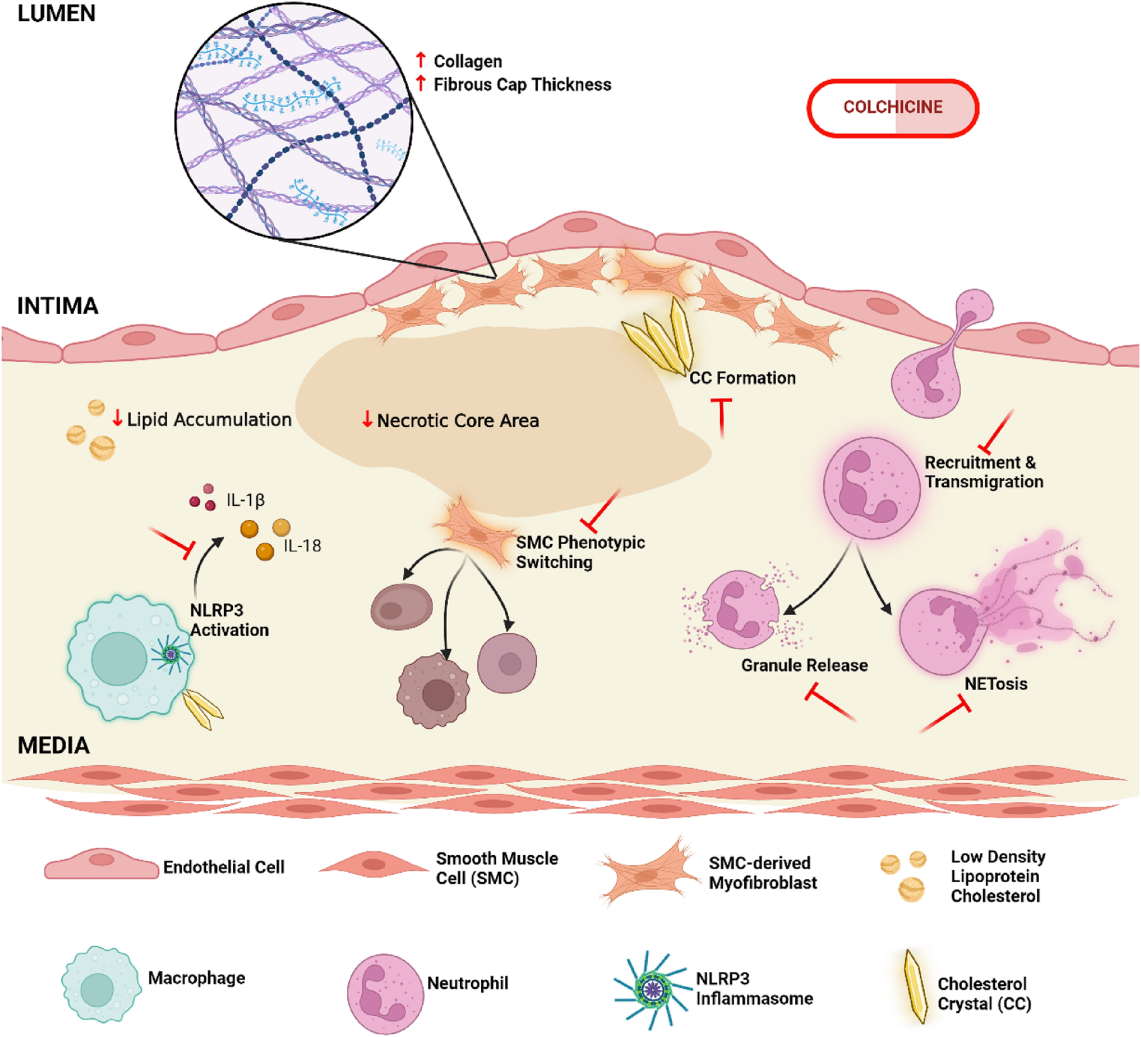
Cellular and molecular mechanisms of colchicine in the modulation of atherosclerotic plaque composition and stability. Colchicine may enhance plaque integrity by promoting collagen deposition and increasing the cap-to-necrotic core ratio, while simultaneously attenuating pro-atherogenic lipid burden. It further inhibits the activation of the NLRP3 inflammasome, a key molecular driver of inflammatory cytokine production, leading to an overall reduction in local inflammatory responses. Other critical aspects of innate immunity are likewise impaired following colchicine administration, specifically neutrophil recruitment to the plaque site and downstream effector functions, including NET (Neutrophil Extracellular Trap)-osis and degranulation. Additional atheroprotective functions include the prevention of vascular smooth muscle cell transdifferentiation from contractile to detrimental synthetic phenotypes, and the modulation of cholesterol crystal formation and morphology; all cumulatively reducing plaque destabilization (created in BioRender.com).

In their review article Bulnes et al. published in this special issue on colchicine in cardiovascular disease, Bulnes and co-workers elaborate on the converging evidence from both animal and clinical studies that show the suppressive effect of colchicine upon the NLRP3 inflammasome, a protein complex that, when activated in inflammatory cells, promotes the production of potent inflammatory, pro-atherosclerotic cytokines, namely interleukin (IL)-1β and IL-18. Cholesterol crystals, which form in the artery wall as the consequence of cholesterol accumulation in plaque, are one such triggering agent for NLRP3 activation.

In their research paper, Abideen et al. detail an inverse relationship between the dose of colchicine used and the formation and expansion of cholesterol crystals *in vitro,* potentially elucidating the drug's indirect effects on inhibiting NLRP3 activation. This is important as there is striking evidence that the accumulation of cholesterol crystal in atherosclerotic plaque can lead to acute plaque rupture (8, 9). *In vitro* assays of rat and rabbit biological membranes viewed using scanning electron microscopy (SEM) showed that cholesterol crystals were able to distort and protrude through the tissue using their sharp geometric edges (8). This was similarly noted in *ex vivo* findings from human arterial tissues and plaques, prepared using vacuum dehydration for SEM, detecting cholesterol crystals disrupting the plaque and penetrating the intimal surface (8, 9).

Abideen et al. provide a novel mechanism by which colchicine may reduce the risk of acute plaque rupture, by showing that it can also slow the rate of cholesterol crystal formation and alter its morphology. Post-drug treatment, SEM of both *in vitro* samples and *ex vivo* samples of human carotid atherosclerotic plaques showed evidence of morphological changes in cholesterol crystals relating to the loss of their sharp edges which, as mentioned, can directly perforate the plaque and trigger atherothrombosis. It remains to be seen if these findings can be replicated *in vivo*.

One of the most recognised actions of colchicine on inflammation, is its inhibitory effect on neutrophil function. However, it is also well-documented to affect the properties of macrophages, endothelium and smooth muscle cells, and dampen the interaction between neutrophils and platelets, which may mitigate against plaque growth, improve plaque stability and reduce the risk of thrombotic occlusion (10, 11). These atheroprotective effects have been exhibited in a mouse model of unstable carotid plaques induced by tandem stenosis surgery, where colchicine was shown to decrease lesion size and necrotic core area, along with increasing collagen and the cap-to-necrotic ratio (10). Interestingly, it has also been recently identified to modulate the transition of detrimental smooth muscle cell phenotypes (11).

Tubulin binding, and subsequent disruption of microtubulin formation, prevents the recruitment and invasion of neutrophils into plaques by disrupting their motility and expression of adhesion molecules. Colchicine further suppresses the formation of neutrophil extracellular traps (NETs): web-like structures released upon neutrophil activation that enhance platelet aggregations and potent cytokine release within the plaque microenvironment (10, 12). A 2021 study by Vaidya et al., showed that colchicine was able to restore cytoskeletal dynamics, and prevent NET formation, in neutrophils of acute coronary syndrome patients post-percutaneous coronary intervention (12). Based on this evidence, Bulnes and colleagues suggest that early administration of colchicine may also modulate initial tissue-damaging responses to ischaemic and reperfusion injury in the setting of acute MI, by preventing detrimental recruitment and activation of innate immune cells.

In another paper in this series, Mohammadnia et al*.* report on a *post hoc* analysis of the effect of colchicine in patients with diabetes from the LoDoCo2 trial. They identified that colchicine resulted in similar reductions in the rate of MACE in people with stable coronary artery disease, irrespective of diabetes status. They also explored whether colchicine might influence the onset of new diabetes and provided weak evidence that it may be protective, which requires further investigation. Indeed, larger prospective trials designed to assess the effect of colchicine specifically in patients with diabetes or on the incidence of new onset diabetes have yet to be seen. Apart from monitoring changes in diabetic treatment and glycaemic control, these studies would need to account for other variables influencing inflammation such as body mass index and diet.

It is recognised that even at low dose, colchicine can cause gastrointestinal symptoms including diarrhea and nausea, however these are typically mild and self-limiting. More importantly, physicians need to be aware that in patients with advanced renal disease or liver failure there is an increased potential for adverse drug interactions, most especially with clarithromycin, cyclosporin and some anti-fungal and anti-viral agents. Those issues aside, low-dose colchicine is safe, as it does not affect renal or liver function, and there have been no reports of drug-drug interactions with other cardiovascular medications, including all forms of statins even when used at high dose (13).

Thus, based upon updated cardiovascular guidelines throughout the world, low-dose colchicine should be considered in patients with high risk of future cardiovascular events based upon a history of MI, stroke or prior revascularisation, as well as those with high coronary calcium scores (14).

Clearly, we have entered a new and exciting era of cardiovascular management in which anti-inflammatory therapy will contribute to the care of patients with ASCVD. Going forward, ongoing and future studies will help determine if there is also a role for newer anti-inflammatory agents with more pathway-targeted actions than colchicine, as these will be needed to treat patients with renal disease in whom colchicine is contraindicated and those who are intolerant to colchicine therapy.

## References

[1] Libby P. The changing landscape of atherosclerosis. Nature. (2021) 592:524–33. 10.1038/s41586-021-03392-8 33883728

[2] Chen W Li Z Zhao Y Chen Y Huang R. Global and national burden of atherosclerosis from 1990 to 2019: trend analysis based on the global burden of disease study 2019. Chin Med J. (2023) 136:2442–50. 10.1097/CM9.0000000000002839 37677929 PMC10586830

[3] Libby P. The forgotten majority. J Am Coll Cardiol. (2005) 46:1225–8. 10.1016/j.jacc.2005.07.006 16198835

[4] Roth GA Mensah GA Johnson CO Addolorato G Ammirati E Baddour LM Global burden of cardiovascular diseases and risk factors, 1990–2019. J Am Coll Cardiol. (2020) 76:2982–3021. 10.1016/j.jacc.2020.11.010 33309175 PMC7755038

[5] Ridker PM Everett BM Thuren T MacFadyen JG Chang WH Ballantyne C Antiinflammatory therapy with canakinumab for atherosclerotic disease. N Engl J Med. (2017) 377:1119–31. 10.1056/NEJMoa1707914 28845751

[6] Tardif J-C Kouz S Waters DD Bertrand OF Diaz R Maggioni AP Efficacy and safety of low-dose colchicine after myocardial infarction. N Engl J Med. (2019) 381:2497–505. 10.1056/NEJMoa1912388 31733140

[7] Nidorf SM Fiolet ATL Mosterd A Eikelboom JW Schut A Opstal TSJ Colchicine in patients with chronic coronary disease. N Engl J Med. (2020) 383:1838–47. 10.1056/NEJMoa2021372 32865380

[8] Abela GS Aziz K. Cholesterol crystals rupture biological membranes and human plaques during acute cardiovascular events-a novel insight into plaque rupture by scanning electron microscopy. Scanning. (2006) 28(1):1–10. 10.1002/sca.4950280101 16502619

[9] Abela GS Aziz K Vedre A Pathak DR Talbott JD DeJong J. Effect of cholesterol crystals on plaques and intima in arteries of patients with acute coronary and cerebrovascular syndromes. Am J Cardiol. (2009) 103(7):959–68. 10.1016/j.amjcard.2008.12.019 19327423

[10] Schwarz N Fernando S Chen Y Salagaras T Rao SR Liyanage S Colchicine exerts anti-atherosclerotic and -plaque-stabilizing effects targeting foam cell formation. FASEB J. (2023) 37(4). 10.1096/fj.202201469R 36856983

[11] Li W Lin A Hutton M Dhaliwal H Nadel J Rodor J Colchicine promotes atherosclerotic plaque stability independently of inflammation. bioRxiv [Preprint]. (2023). 10.1101/2023.10.03.560632

[12] Vaidya K Tucker B Kurup R Khandkar C Pandzic E Barraclough J Colchicine inhibits neutrophil extracellular trap formation in patients with acute coronary syndrome after percutaneous coronary intervention. J Am Heart Assoc. (2021) 10(1). 10.1161/JAHA.120.018993 33346683 PMC7955504

[13] Nidorf SM Ben-Chetrit E Ridker PM. Low-dose colchicine for atherosclerosis: long-term safety. Eur Heart J. (2024) 45(18):1596–601. 10.1093/eurheartj/ehae208 38596868

[14] Virani SS Newby LK Arnold SV Bittner V Brewer LC Demeter SH 2023 AHA/ACC/ACCP/ASPC/NLA/PCNA guideline for the management of patients with chronic coronary disease: a report of the American Heart Association/American College of Cardiology joint committee on clinical practice guidelines. Circulation. (2023) 148(9). 10.1161/CIR.0000000000001168 37471501

